# Metaphorically speaking: considerations in applying metaphor to your research higher degree

**DOI:** 10.1177/17449871251403373

**Published:** 2026-01-20

**Authors:** Amy Byrne, Jennifer Mulvogue

**Affiliations:** Senior Lecturer and Postgraduate Research Coordinator, School of Nursing, Midwifery and Social Sciences, CQUniversity, Sydney, NSW, Australia; Head of Course Mental Health, School of Nursing, Midwifery and Social Sciences, CQUniversity, Brisbane, QLD, Australia

**Keywords:** education, language, learning, metaphor, research higher degree, thesis

## Abstract

**Background:**

The use of metaphor in qualitative research higher degrees can be a creative and effective outlet. Not only can metaphor make connections across concepts, but it can help to create a coherent theme within a wider body of research.

**Aim:**

This paper provides a ‘how to’ of sorts, providing a pragmatic guide for the use of metaphor in research work, particularly in research higher degrees.

**Methods:**

Drawing from contemporary metaphor theories, the paper articulates eight steps to assist users in the contemplation and application of metaphor in research.

**Results:**

This paper provides not only a thought-provoking process for candidates and researchers to apply metaphor but also offers a pedagogical tool for supervision teams and research institutions to support learning in qualitative research methods, bolstering critical thinking, communication translation, and academic writing.

## Introduction

A metaphor is a figure of speech, a representation and/or a comparison of two things that on the surface have unrelated features but have some associated qualities (Jensen, 2019). Metaphor is a powerful tool that assists writers to express complex phenomena to the reader. Facilitating connection and understanding, metaphor is often adopted in research to draw parallels between outcomes and everyday understandings. The metaphor creates a coexistence between two concepts allowing interdisciplinary thought, cognitive content, and exploration (Kelly, 2011).

Metaphor is a useful tool to analyse the many dimensions of complex concepts or complex systems (Abawi, 2013). Durkee (2011) described metaphor as being used in both undergraduate and graduate courses, as a method to teach critical thinking skills. The impact metaphor has on the imagination may help a student to understand concepts in synchronicity between reality and abstract conceptualisation (Guilherme et al., 2018). Indeed, the application of metaphor is reciprocal – with both the writer making expression, and the reader making meaning.

The Research Higher Degree (RHD) journey can be a life-changing process (McIntosh, 2015) and grasping complex theoretical knowledge can be difficult. Thesis work is a social practice which often applies a broad discursive context. Metaphors can be used in education and in qualitative research to explore abstract or speculative ideas or used as a framework to help make meaning of the work (Abawi, 2013). Supervisors may assist RHD candidates by initiating and encouraging creative thinking in candidates through inventive learning modes. The metaphor brings with it sociability, connections through meanings, and an association to the outside world and its relevance to the research (Kelly, 2011). Doctoral creativity plays an essential role in extending knowledge boundaries; however, this is not always encouraged, despite the process being found to extend the field of knowledge (Frick, 2020). The hesitation to promote creativity may stem from the longstanding challenge qualitative research faces in being seen as equally credible as quantitative research. As Cardona (2020) explains, qualitative research is often considered ‘invisible’ because it explores phenomena that cannot be directly observed, unlike quantitative research, which focuses on clearly measurable and observable data.

While metaphor is a well-known and often used tool in research, why and how it is applied is not always clear; thus, this paper aims to provide discussion around its use and provide conceptual and pragmatic examples that qualitative researchers may consider.

The aim of this paper is to develop capability around metaphor use in research, particularly in qualitative and critically positioned inquiry. In doing so, this paper provides a ‘how-to’, for RHD candidates or any researcher looking to apply metaphor.

## Metaphor theory background

Contemporary theory stems from the work of Reddy’s (1979) *conduit metaphor*, which demonstrated that everyday English language is laden with metaphor and acts as a way for us to understand and conceptualise the world we live in. Although metaphor is expressed through language, it is, importantly, a part of our thought processes, intrinsically linked to the way we interpret what is around us (Reddy, 1979). Metaphors are thus more than words; the language is ‘secondary’ to a mapping of a conventional systems that we understand as daily life (Lakoff, 1992, p.6). Lakoff (1992) described metaphor theory through generalisations, motivations, categories, invariable principles, event structuring, duality, and much more.

From a theoretical perspective, Conceptual Metaphor Theory (CMT) provides a basis for the use of metaphor in research. Conceptual metaphor is defined as ‘understanding one domain of experience (that is typically abstract) in terms of another (that is typically concrete)’ (Lakoff and Johnson, 1980: 112–113). As described by Lakoff and Johnson (1980), CMT describes metaphor as more than ornamental, but rather a tool for structuring, restructuring, and *creating* reality (Kövecses, 2020). Throughout CMT, metaphor is believed to be all pervasive, including in deliberate and non-deliberate use, as part of the linguistic vernacular. This is evident through media, conversations, dictionaries, and many forms of discourse. Metaphors are also modalities in mapping knowledge, moving from concrete to abstraction thought (and vice versa), which act to create and (re)construct reality as we understand it (Kövecses, 2020).

Tourangeau and Sternberg (1982: 205–206) described the composition of metaphor as the ‘tenor’ or ‘subject’ bearing some resemblance to something else, but that there is always some dissimilarity. That is, there is never an exact comparison, more that it is grounded in a shared category or has shared membership features. An example is the phrase ‘water off a duck’s back’, which carries both literal and metaphorical meanings. Literally, it refers to how water effortlessly rolls off a duck’s feathers, leaving the duck seemingly dry or unfazed. Metaphorically, it describes a person who easily shrugs off criticism or negative remarks.

The surface form of the metaphor is linked to a deeper structure or explicit comparison, of conceptualised literal frames and the transferring of the metaphorical elements into canonical configuration, where the underlying comparisons are applied. Therefore, they may involve a complex interpretive process that needs to be more deeply understood (Tourangeau and Sternberg, 1982).

The concept of metaphor dates to Aristotle’s Poetics, where he defined it as ‘giving the thing a name that belongs to something else’ (1902: 21). He saw metaphor as a powerful rhetorical tool, used by poets to delight and by politicians to persuade (as cited in Alharbi, 2023). Over time, metaphor methodology has expanded into various branches. In the 1920s, philosopher José Ortega y Gasset explored gestures and emotive expressions through a phenomenological lens, identifying the need for dialogue between phenomenology and cognitive linguistics. This interdisciplinary connection was later realised by Lakoff and Johnson (previously discussed), who introduced the theory of conceptual metaphor in the 1980s (Expósito et al., 2024). In contemporary times, metaphor is used extensively as a method across diverse theoretical frameworks.

This is in no way an exhaustive description of metaphor theory, which has evolved over time and social space, but instead provides a theoretical footing for this paper. Additionally, it is important to note that metaphor methodologies, as a form of analysis, are an important research tool. This paper does not attempt to provide any guidance/commentary on such methodologies and instead focuses on a pragmatic guide for researchers to apply when considering metaphor use. We encourage researchers to explore metaphor theory and analysis as a separate and important endeavour to its application.

## Methods

This paper was created to support RHD students and researchers by offering a practical guide for applying metaphor in research.

This paper leverages the methods described by Byrne et al. (2025) in articulating a stepped process for research development and presents literature, along with reflective questions and examples to guide the reader through the application of metaphors in research. It allows users to reflect upon, adopt, and apply metaphor to their work.

To determine the steps applied within this paper, the authors consulted the literature and reflected on their own use of metaphor in research, leveraging their experiences as candidates and supervisors. A series of meetings were held, where the authors reflected upon the use of metaphor, how RHD candidates and researchers use metaphor, and the steps required to provide clarity on this. Based on these reflections, draft steps (acting as both background information and pragmatic processes) were created. These steps were reflexively refined throughout the writing process and engagement within the literature. This resulted in eight steps for the application of metaphor. Reflective questions and examples are provided for pragmatic application.

## Results

Eight key steps are presented:

1. Why use a metaphor?
2. Finding the right metaphor.
3. Metaphor to express power and ideology.
4. Metaphoric imagery.
5. Sowing a common thread – linking ideas across the research.
6. Metaphor as a method of signposting.
7. Metaphor as a culmination and catharsis.
8. Implications – skewing results and/or connecting and deeply learning?

### Step 1. Why use a metaphor?

Metaphor is a powerful tool to make meaning of complex situations and phenomenon. While the focus of this paper is qualitative research, metaphor can and is applied in quantitative paradigms: Taylor and Dewsbury (2018: 2) provided examples such as research ‘blueprints’ or ‘recipes’, analogies which facilitate the processes, design, and outcomes of research.

In critical paradigms, metaphor allows socio-political messages to be uncovered, expressed, and debated. For example, describing situations as a ‘war’ allows militaristic strategies to abound in the combating of issues (Taylor and Dewsbury, 2018, p. 2). It may be used to describe initiating or encouraging action, as seen in Mainey et al. (2025: 1), where the metaphor of ‘awaking the sleeping giant’ was used to describe the unrealised or potential political power of nurse advocacy.

In qualitative research, storytelling has a profound impact on readers, evoking emotions and emotional connections to the subject matter. As such, the gravity of research outcomes and recommendations can be highly supported through metaphor (Carpenter, 2008), particularly where they are social and emotional in nature. Metaphors often reflect to the reader the ways that people think, act and communicate, creating connections and insights into societal problems (Redden, 2017). For example, Barnard (2025, p. 87) who studied non-suicidal self-injury, used the metaphor of ‘barriers and bandaids’ to describe external and systemic factors preventing health consumers from experiencing therapeutic interactions. The fragile nature of solutions or treatment interventions is thus highlighted.

Reflective question and exercise:

1. How might metaphor assist you to tell the story of your research?
2. In what way will a metaphor assist in creating meaning and impact?

### Examples

In Mulvogue’ s (2024) thesis – *Machinations of restraint in residential aged care facilities*, critical discourse analysis was employed to examine how chemical and physical restraints are used in aged care settings. Concepts of political rhetoric, the meaning behind political speak, and how words are often washed clean and convoluted were raised. As such, Alice in Wonderland offered a poignant way of driving the message.

In Alice in Wonderland, the White Rabbit reads a non-sensical statement aloud to the court. When Alice queries what it means, the King reassuringly advises the court room not to worry about it, that it is better to accept it, even if it is not understood. Parallels between this scene and political discourse, where text is complex, the meanings often hidden through strategy or language, are drawn.

‘If there’s no meaning in it’, said the King, ‘that saves a world of trouble, you know, as we needn’t try to find any’. (Carroll [Alice in Wonderland], 1865/2018, Chapter 4, p. 113).

In exploring the metaphoric connections between the fictional court scene in the Alice in Wonderland story, Mulvogue was able to identify similarities in language use, euphemisms, political speak, rhetoric, and creating deeper critical learning and linkages in her own work about Australian aged care reform.

### Step 2. Finding the right metaphor

With the understanding that metaphor can be used to express, make meaning, and form connections, the next step for researchers may be to consider which metaphor might be right for their research. Tourangeau and Sternberg (1982) described various ways in which metaphors can be used. One view is that metaphors compare two terms with just enough insufficient literature comparison. For example, ‘A wild goose chase’ invites the reader to imagine a pointless or meaningless endeavour. Tourangeau and Sternberg (1982) noted that critics of the comparative view argue a different viewpoint; the metaphor points out the anomaly rather than the comparison; it is the obvious dissimilarities between the two terms or subjects that creates a form of absurdity. For example, the commonly used metaphor ‘The elephant in the room’ suggests a level of ridiculousness, an unlikely scenario where an elephant in a room would not be spoken of. Yet the meaning of the metaphor is that the ‘elephant’ resembles ‘a large issue’ not addressed. This metaphor uses more absurdity than comparison.

The use of metaphor is useful to stimulate emotion, reaction, or to motivate deeper learning. Lubart and Getz (1997) proposed that emotions generate metaphors for critical thinking and that humans attach emotions to images or concepts. The way they are interrelated can unleash creative associations in memory elements, with the metaphor providing means to express emotional concepts that aid problem-solving. Finding the right metaphor may rest on the emotion behind the research. The use of metaphor may guide the researcher to direct these emotional concepts into critical problem solving.

It is important, therefore, to consider the purpose of the metaphor. It should have a level of familiarity, symbolism, process, or abstract conception to enable connections to be made. There is generally a level of reality versus imaginary and emotion versus logic. The choice of metaphor is often a very personal one, though it may also be found through reading or created by a team.

Metaphor is a tool used commonly in the English language. Often, researchers use metaphors sporadically through text in a situational manner (whether intentionally or unintentionally). At other times, it is used purposefully and is extended throughout the body of work. Given this, researchers will naturally have more than one metaphor. Researchers might consider an overarching or extended metaphor which unifies and drives the research, along with more nuanced metaphors which apply to specific concepts.

Reflective question and exercise:

3. How will the metaphor evoke meaning and emotion (or how will it not)?
4. How did the emotion or the movement within your research influence your thinking about the metaphor you considered?
5. What is the main message you want to convey, and how would you describe this to a lay person? What metaphor best assists in delivering the message?

### Examples

In Byrne’s (2022) thesis – *Person-centred care as a technology of compliance*, the metaphor of music and symphony was used. The thesis was a critical evaluation of how the concept of person-centred care is delivered in the neo-liberal landscape, and how multiple singular elements come together in a larger amalgamation of processes and workflow for the provision of care. The metaphor of a symphony of music was discovered through the work of Povinelli (2011), and was adopted to demonstrate the influences, history and power, as exampled in Byrne’s work (2022, p. 91).

In her work Economies of Abandonment, Povinelli (2011, p. xvi) described and mapped the influences of liberalism as a ‘symphony’ to track the movements and ideologies of government markets in relation to each other (Khalidi, 2017). . ..My [Byrne] symphony represents a conceptual approach to identifying touch points of power that may or may not have a direct influence on how Person Centred Care is interpreted, established, and enacted. . .

**Figure 1.**
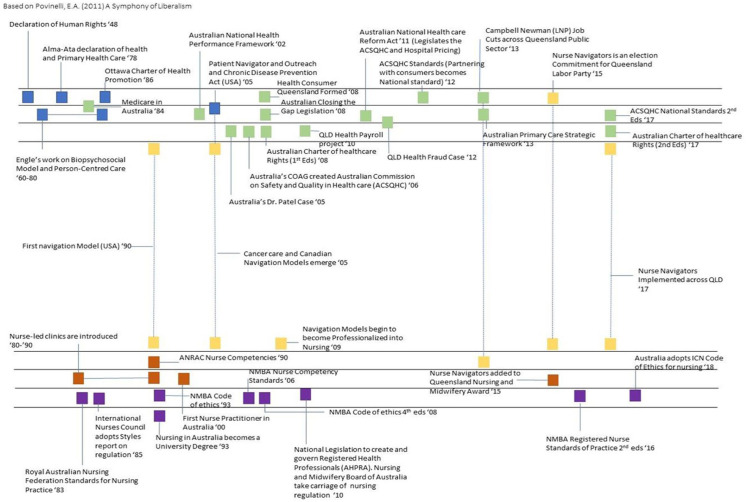
‘Symphony of liberalism’ – Example of how the metaphor can be applied (Byrne, 2022).

In this case, music is abundantly understood, recognised, and consumed in society. Music itself is made up of individual notes, chords, bars, and stanzas, which come together to form a melody, and more broadly, the symphony of music. In Figure 1, a visual metaphor captures the alignment between person-centred care and a ‘symphony of liberalism’.

### Step 3. Metaphor to express power and ideology

Goatly (2006: 25) posited that metaphor is a tool of ideology, acting to create ‘meaning in the service of power’. Goatly (2006) suggested that metaphors in themselves perpetuate understood norms and values, particularly in relation to power, and can reinforce hierarchy, racial divides, and other conflicts. Steen (2011) suggested that metaphor, through their use, go through several transitions – from metaphor to thought, and from metaphor to language, thought, and communication. Thus, metaphors are a powerful tool, and in themselves, a *power* tool.

Further demonstrating this, metaphor is commonly used in political discourse, allowing governments to express messages in ways that suit their agenda. Filipczuk-Rosinska (2006) found that much political discourse used war themes (e.g., shooting down an argument, attacking an opponent), imparting a somewhat violent ideology to the power within politics.

Similarly, researchers may adopt metaphor to assist them in highlighting forms of oppression and power, and use it as a form of resistance, calling out the inequity or absurdity of modern structures. For example, the metaphor of a kingdom, monarchy, and oligarchy may be adopted to demonstrate rule over all and the concentration of wealth. It might also be used to demonstrate that power can only exist within a relationship, such as the sonnet written by the poet Percy Bysshe Shelley written in 1817, ‘Ozymandias’ (the alternate name for the Egyptian pharaoh Ramses II). The poem describes the crumbled statue of Ozymandias in a foreign desert, which stood as two vast stone legs and a buried head in the sand, with the inscription ‘King of Kings’, describing the king’s achievements as invoking awe, and despair in others. The inscription and ironic corroding reality of the ruins of the statue underscoring the transient nature of political power (Lit Charts, n.d.). It exposes the stark reality that the once-mighty statue of the ‘King of Kings’ lies in ruin in a remote desert, symbolising how even the greatest power is ultimately vulnerable to the relentless passage of time (Lit Charts, n.d.).

Metaphor use has vast application for the researcher in its utility to broaden, deepen and bolster the critical thinking of a researcher. In critical work, the researcher studies semiotic dimensions of power, of politico-economic cultural changes, and societal injustices; and analyses rhetoric, anthropology, and philosophy (Wodak, 2014). In a way, researchers themselves are enacting what power they have, and metaphor allows for the translation of ideas, the emancipation of people, and broader advocacy.

**Figure 2.**
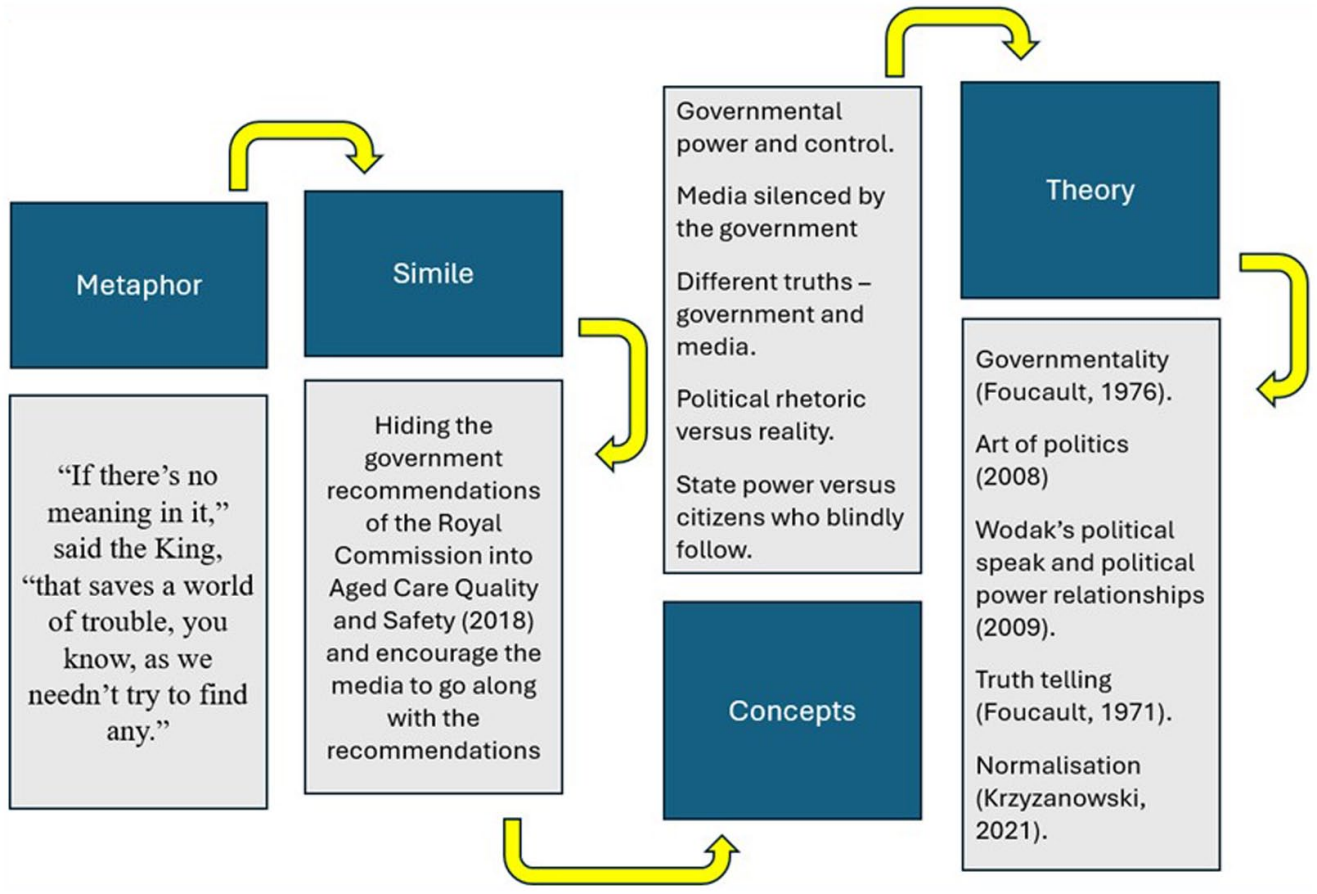
Connecting the metaphor to power. Source: Created by the authors.

Consider the following concept map describing the travel of hegemony within the court scene of *Alice in Wonderland* (Carroll [Alice in Wonderland], 1865/2018, Chapter 12, p. 113) in Figure 2. Points from the metaphor can be used to extract and explore parallels. The example below was created using Mulvogue’s (2024) doctorate. As described below, the metaphor can traverse similes, concepts, and theories within the work, drawing out power.

The quote from Alice in Wonderland (see Figure 2, far left) has parallels with features to a media release of the recommendations of the Royal Commission in Aged Care Quality and Safety (Government releases final report of Royal Commission into aged care, 2021). Questions from the media were dismissed as minor issues. This association linked to concepts, such as government silencing media, government control and rhetoric, political ‘truth telling’ may be linked to theories, such as normalisation, governmentality, and political speak.

Reflective question and exercise:

6. What elements of power and/or ideology are evident in your research?
7. What are the similarities of historical versus contemporary hegemonic structures?
8. What metaphors may be used to move between historical and contemporary societal concepts or thinking?

### Examples

Themes of war are common in everyday language, and as suggested above, abound in political discourse. When in agreement, the political leaders may be said to have ‘drawn a truce’ or agreed to a ‘ceasefire’. Byrne (2022: 231) also used a metaphoric example in her thesis: ‘A fiscal and ethical war within healthcare’. This highlights that economic management of health services is often opposed to the ethics of care, that is, biopsychosocial models are timely and costly but are also effective and deeply regarded by individuals. Discursively, these two ideologies are often played against one another to drive whatever agenda suits.

Mulvogue created metaphors drawing from the work of Foucault (in *Discipline and Punish*, 2007) to describe how the state (government power) uses the human body much like a soldier, and intentionally shape and discipline it to use it as a resource. The fighting body is used to be ‘productive’ in much the same way that people in aged care facilities have their bodies medicated, ultimately rendering them docile and subdued, yet their bodies are ‘productive’ because they generate income for the institution.

### Step 4. Metaphoric imagery

Metaphors are not just expressed verbally or in textual language; they can also be expressed through imagery. Textual metaphors often give rise to visual perceptions and understanding (Carston, 2018), for example, a poem which used the metaphor of a person walking down a road may evoke this imagery in the reader. Mental imagery is essential to understanding a metaphor’s use, helping to form connections and meanings (Garello, 2024).

However, a tangible image in research can also be applied, provide a visual prompt to the reader, breaking up large sections of writing and (un)consciously remind the reader of the deeper metaphoric thread being weaved within the research. Ventalon et al. (2023) explored the use of visual metaphors and depicted images of scales (a school bag and a cinder block on each scale), contrasted images of a person with a line down the centre of their face – on one side a nurse, on the other an angel. Their work demonstrates the juxtapositions, relationships, hybrids, personification, and contextual relationships that visual images can take. This has important implications for researchers.

**Figure 3.**
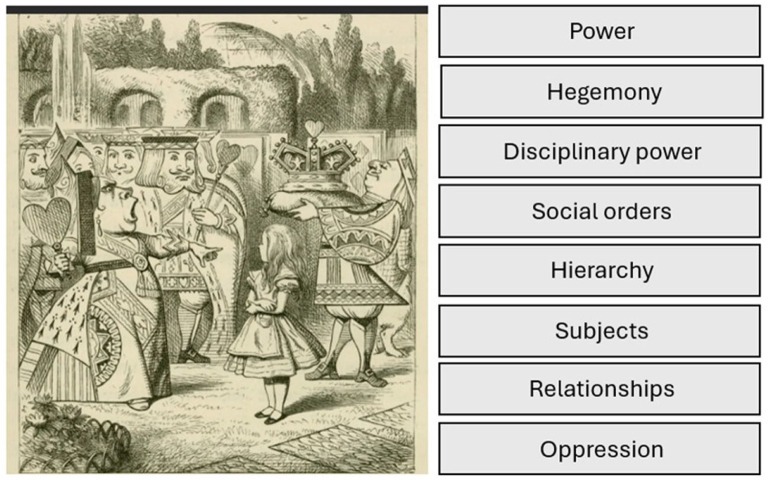
Consider Alice and the croquet scene. Image (left) from Tenniel (1967). Used under creative commons copyright.

The metaphoric use of text and image to demonstrate layers and intricacies of power can have impact for researchers. An example of power can be clearly identified in the Queen of Hearts (Carroll, 1865) image below. Indeed, such an image could be interpreted as depicting the hidden power often described in critical studies and the various sources of hidden and oblivious power woven through discourses. Figure 3 prompts the reader to consider the image in relation to power under a critical lens. Readers may want to adapt the prompts to suit their specific paradigms.

Reflective question and exercise:

9. Can you identify a metaphoric image to use in research? What would it look like?
10. What will imagery evoke to your readers? How will it assist in making meaning?

### Examples

**Figure 4.**
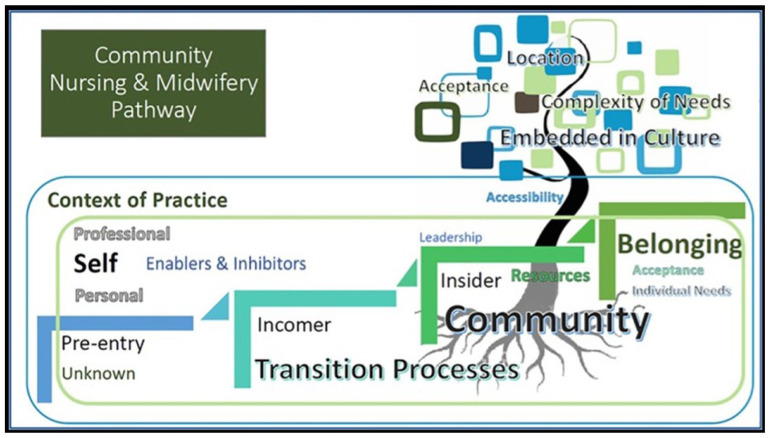
Community nursing and midwifery pathway ‘Tree’. Source: From Harvey et al. (2019; used with the permission of the corresponding author).

Harvey et al. (2019) developed a community-based nursing and midwifery pathway through qualitative research. The research used the imagery and metaphor of a tree to signal growth. Indeed, this imagery was used to then develop a more applied research framework for the development of nurses and midwives. The image used the tree to demonstrate strong foundational knowledge (roots of the tree), accessibility of the pathway (tree trunk) and the outcomes (leaves). This is demonstrated in Figure 4.

### Step 5. Sowing a common thread-linking ideas across the research

A good story relies on a solid plot, and a consistent story thread which helps to connect scenes, people and places (among many other elements). Metaphors often assist in framing the narrative, giving them a specific function in research (Semino et al., 2018). Indeed, metaphors can set the theme and convey the meaning and emotion of a story. For example, Johnson et al. (2023) explored metaphors in pain, where stabbing and gnawing provide themes of violence and attack, where the person is a victim of pain and socially positioned as such.

In this same vein, careful consideration must be given to the extended metaphor, and what emotions, ideologies and messages it delivers. For example, one must avoid trivialising the research through the metaphor. Indeed, audience is important; medical research for example has found that the metaphors used in consultations can change based on the interactional dynamics (Declercq and van Poppel, 2024). Researchers may also consider the use of metaphor within their field, consider their likely audience and the gravity and importance of the research.

Students and researchers can use metaphor in various ways. As mentioned above, metaphor use is often second nature and unconscious; however, its use to make deep, reflective linkages in research is particularly powerful. Bringing the research together throughout what is known as a red or common thread is key. Edwards-Groves and Ronnerman (2021) posited that the red thread within a narrative provides flow and interconnectivity; the red thread itself being a metaphor for a cohesive narrative that is present throughout a story. In this way, metaphor can be used as the thread or to assist in communicating ideas throughout. For example (and using some of the metaphors we have evidenced in this paper), the research may best be described as a journey down a road, as a wheel with many cogs, as a stage with actors upon it, as an iceberg looming under the surface.

Reflective question and exercise:

11. How will your metaphors be applied across the different areas of your research?
12. How will you use metaphor to make connections for the reader and provide cohesion throughout your document (for example thesis)?
13. How will your audience understand this research? Does the metaphor do justice to the work?

### Examples

Consider the metaphor ‘All the world’s a stage’ – a famous quote from the Shakespearean play *As You Like It* (Shakespeare, 1623).

All the world’s a stage,

And all the men and women merely players;

They have their exits and their entrances;

And one man in his time plays many parts. . .

This script was conceptualised and written in the time of the English renaissance and the early modern era, through a landscape of battles for kingdoms and revolution – a time when the English population became more aware and influenced by literature, art, and architecture (Douglas, 2018: xvii). It was an era of great exploration, creativity and changing perceptions around religion, of wars and discoveries of new lands, increased social mobility, and advances in economic, cultural, and political possibilities (Douglas, 2018: xvii). Arts reflected the culture of the times, so it is interesting to wonder what had prompted Shakespeare to develop the worldly and widely used metaphor at this time. Later, in the 20th century, the scholars of the Frankfurt School were exploring themes, and deliberating socialism, Marxism, sociology and philosophy, and the development of critical theory philosophies (Garlitz & Zompetti, 2021). The concepts of Shakespeare’s play align with concepts of society, of ‘actors’ playing a role. In the 1970s, the metaphor again rang true, through Foucault’s lectures and discussions on governmentality, people’s place in society and its relationship to government, the economy and individual freedom and artificially arranged liberalism (Foucault, 2008; Lemke, 2001).

All the world’s stage provides a potential common thread to research. One might link the participants in their study to ‘actors’ on a stage, ‘merely players’. The institution, organisation or social epoch that they belong to are the ‘stage’. The performative nature of the stage may also be leveraged, as people have their defined ‘exits and entrances’. Other motifs may come into play, such as the opening a closing of a curtain, acts within a play, audiences, entertainment and much more.

### Step 6. Metaphor as a method of signposting

Signposting (which is, ironically, a metaphor in itself) is a process of navigating the reader throughout the thesis and/or research. Through signposting, the author gives signals, reminding the reader of the importance or relevance of certain aspects, within the research. Signposts can be used in various ways; some people do this textually, giving the reader fair warning before they arrive at a certain part. For example, ‘in this section I discuss the differences between honeybees and bumblebees’.

Academic work often rests on historical conventions and follows similar structures, for example research papers and thesis work tend to follow well-known and familiar conventions, dictating the order of the work, such as where the introduction is positioned, where the methods are and what should be included, such as indices of tables (Malone, 2023). The conventions are historically tied and deeply socially significant. Malone (2023) argued that such conventions based on academic or expert research are often presented as the ‘only way’, thus potentially reducing student learning to predefined constructs.

As such, metaphorical signposting may provide opportunities for candidate creativity while serving a cognitive function for readers. Metaphors can be used to remind the reader of the commonality throughout the work, drawing the reader in subtle ways back to the extended metaphor. For example, a road, a path, a driver, a dirt road, a highway, all serve to remind the reader of a journey metaphor but may also signpost uncovered issues (a dirt road) or common schools of thought (a highway).

Reflective question and exercise:

14. How will your metaphor be used to signal to the reader what is to come?
15. How could you use metaphor assist to signpost your research?

### Example

**Figure 5.**
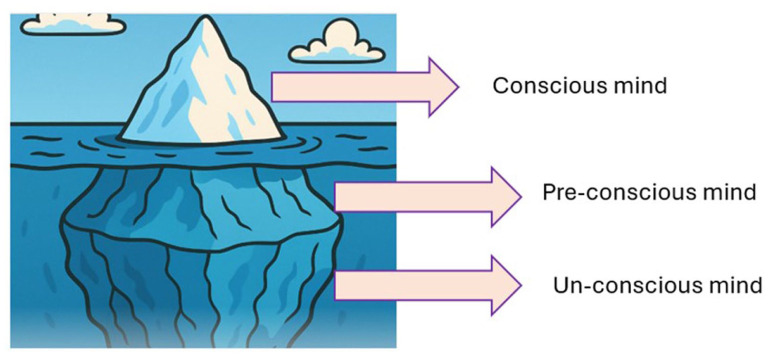
The Iceberg Model This image was created with co-pilot image generation, based on the work described by McLeod (2024).

Freud used the iceberg metaphor to explain the structure and the function of the brain over three levels: the conscious, preconscious, and the unconscious. Freud noted that, like an iceberg, most of the mind lies under the surface, hidden and unseen. McLeod (2024) used this image to express meaning and to scaffold learning. The metaphoric imagery is used below (Figure 5) as a signpost, and the arrows and sub-sections were used as headings, directing the reader to what is to come. Due to copyright, we have replicated the imagery described by McLeod (2024), demonstrating how it can be used to simultaneously connect thoughts and to signpost (scaffold) what content is to come.

### Step 7. Metaphor as a culmination and catharsis

The final step in considering your metaphor is in how it might be used to draw conclusions, climax the research, and act as a catharsis. Conclusions from research are often driven by complex consideration and framing, where the research comes to clear outcome(s) through analysis. Culminating the research, particularly in lengthy RHD work, is essential, and bringing the work to a high point may be multifactual.

Metaphors hold the ability to build an argument, and to provide flexibility and creative freedom. Jamrozik et al. (2016) posited that metaphors can be used sparingly before being elaborated on as the base of knowledge is broadened. Different types of metaphors lend themselves to this process; for example, attributive metaphors (the moon is an orb) share object similarities, and the application of such a metaphor may not extend beyond descriptive, analogous means. On the other hand, relational metaphors (cogs in a machine, the battlefield) allow for more nuanced connections to be formed, and meanings to be conveyed (Jamrozik et al., 2016). Again, a mix of different types of metaphor are likely (given the unconscious way we often apply them), yet an extended metaphor may hold the work together and bring it to a close.

Indeed, metaphors can be cathartic. Stanley et al. (2021) explored the use of metaphor in people who had a lived experience of the COVID-19 pandemic in the United States. The researchers explored contextual sense-making about their metaphors, revealing participant mental models including danger, uncertainty, misery, grief, anger, disgust, and fear. This paper has demonstrated that metaphor allows connections to be made across the continuum of research. Similarly, metaphor may provide opportunities for researchers to release the findings and toil of the work, making conclusions meaningful to readers and to themselves (Steele et al., 2022).

Reflective question and exercise:

16. How could the metaphor work to culminate your research? Think about the flexibility and transferability of the metaphor, particularly as findings may not yet be known.
17. How might your metaphor assist in supporting you to translate, and move the research forward? Think about any future applications of research.
18. How could your metaphor assist you in coalescing your own research journey?

### Example

**Figure 6.**
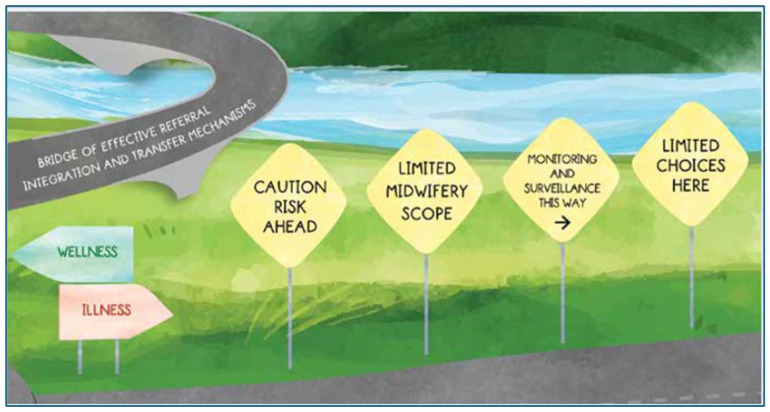
Bridge to effective referral. Source: From McNaughton et al. (2022; used with the permission of the corresponding author).

**Figure 7.**
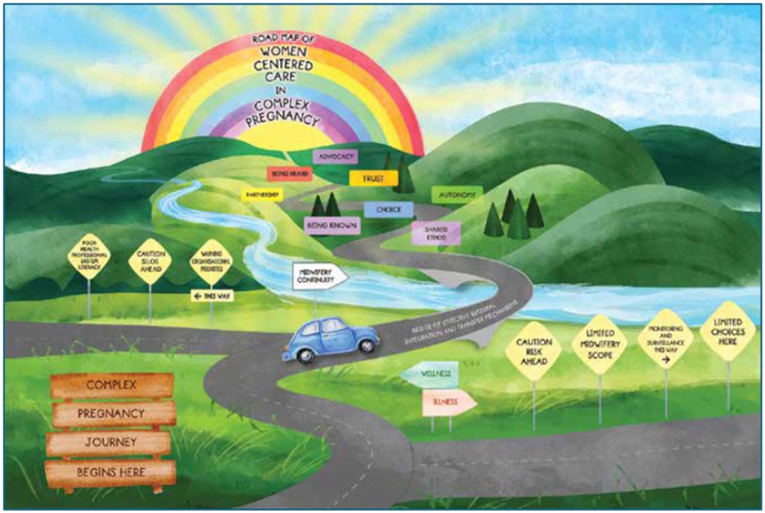
Road map of women-centred care in complex pregnancy. Source: From McNaughton et al. (2022; used with the permission of the corresponding author).

In their work on midwifery and the provision of women-centred care, McNaughton et al. (2022) used the metaphor of the journey, and as such the road was a strong motif. A consistent thread was used to demonstrate the journey and to convey findings and meaning from the research. Figures 6 and 7 demonstrate this, while also depicting a culmination of the research in its entirety.

While this example was achieved using imagery, other forms of discourse and motifs can be used in forming conclusions, including the use of text.

### Step 8. Implications – skewing results and/or connecting and deeply learning?

Metaphors serve many purposes, particularly in clarifying abstract or complex ideas. Unlike everyday language, which relies on familiar and concrete terms, metaphors offer a way to express nuanced concepts (Yildiz and Dede, 2025). However, their use in research must be carefully considered. In certain contexts, metaphors may be inappropriate or misleading. It is essential to place them thoughtfully, where they enrich understanding and invite the reader to reflect on the layered meanings they evoke, in appropriately placed areas of research papers.

Metaphors, while powerful tools, must be used with care. Their placement within research is crucial. As Manhas (2015) noted, metaphors serve as linguistic and conceptual aids that can shape analytical patterns or support recontextualisation. However, they also carry the risk of distorting research findings, particularly the lived experiences and meanings expressed by participants. Researchers must therefore ensure that metaphoric language does neither compromise the dignity and integrity of participants nor allow aesthetic appeal to overshadow the reality being represented.

We must thus consider if metaphor use expands beyond research to our very own humility, our ability to connect meaningfully and to feel emotions. A study by Fetterman et al. (2020) found a link between days that a person felt more empathy to higher metaphor use. The researchers found a link between metaphor use and connectivity to emotions and perspectives of other people. Therefore, metaphor use has purpose not only in research but also in our communication and connection to ourselves and others.

**Figure 8.**
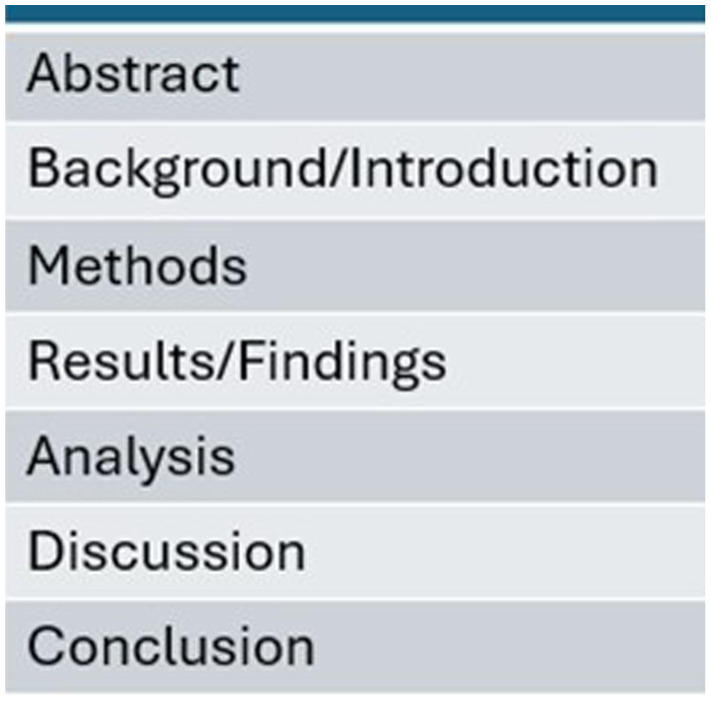
An example of a research paper’s skeleton template.

Reflective question and exercise:

19. Considering the table below, which is a basic skeleton template of a research paper, which areas would you consider metaphor more likely to be useful and meaningful? And which areas would you use more caution (Figure 8)?

Fetterman et al. (2020) found a link between days that a person felt more empathy to greater metaphor use. Spend some time over the next couple of days listening to the metaphors you use in your everyday language and then consider how these metaphors are connecting your thoughts, understandings, and emotions.

## Discussion

There is no specific person who will benefit from using metaphors, more than another. Some will gravitate to metaphors as a form of pedagogical learning, and others will need assistance in their consideration and application. Whatever the case, it is important to find tools to advance higher order thinking in RHD candidates, or indeed any form of research.

The use of metaphor in research extends beyond its epistemological and methodological utility; it also holds significant pedagogical value (Lynch and Fisher-Ari, 2017), particularly in the context of RHDs and academic supervision (Mouraz et al., 2013). As Waring and Evans (2015: 27) described, pedagogy is about choices, in what things are taught, how and why: reflective of a social agenda, where power relationships exist and empowerment is framed around these relationships. It may not only be a question of the right metaphor fit for the research, but also about how it is perceived, how it relates to the student, whether it is useful in connecting complex systems to narratives and evoking new ways of thinking. As this paper has demonstrated, metaphor is not merely a literary flourish, but a powerful cognitive and communicative tool that can support learning, critical and creative thinking, and scholarly identity formation (Ivie, 2022). These qualities position metaphors as a valuable strategy, especially in disciplines that engage with complex, abstract, or emotionally charged content.

Metaphor facilitates learning by enabling students to connect unfamiliar or abstract research concepts with familiar, concrete experiences (Ivie, 2022). For example, when a candidate likens their research (including their own journey within research) to navigating a labyrinth or composing a symphony, they are not only expressing their experience but also engaging in a form of conceptual mapping that deepens their understanding of the research process (Steen, 2007). Metaphors can help scaffold learning by making the invisible structures of research more visible, relatable, tangible, and tactile.

In the supervision of RHD candidates, metaphor can serve as a dialogic tool that fosters reflective practice and epistemological awareness, cultivating wisdom and understanding, and strengthening the supervision relationship (Scobie et al., 2021). Supervisors who encourage the use of metaphors invite students to explore the emotional and intellectual dimensions of their research. This promotes a more holistic engagement with their work (Hanić et al., 2018) and can be particularly valuable in qualitative and critical paradigms, where the researcher’s positionality and interpretive lens are central to the inquiry. Metaphor thus becomes a means of articulating and negotiating the often-ambiguous terrain of doctoral research, supporting students in developing their scholarly voice.

In writing this paper, we aimed for pragmatic and adaptable utility. The reflective exercises and eight-step framework can be adapted for the individual’s context. The process may also be adopted for use in supervision meetings, research training programmes, thesis writing workshops and postgraduate coursework. These pedagogical framings offer a structured opportunity for candidates to explore metaphors as a tool for meaning-making, narrative cohesion, and knowledge translation. For instance, asking students to identify a metaphor that encapsulates their research questions or findings can prompt deeper engagement with their topic and foster creative thinking.

Additionally, metaphors also play a role in knowledge dissemination. By translating complex research findings into accessible, metaphor-rich narratives, researchers can engage broader audiences and enhance the societal impact of their work. This is particularly relevant in fields such as health, education, and social sciences, where the ability to communicate research in compelling and relatable ways is essential.

We strove to develop this paper in an inclusive way, allowing teams and individuals to consider themselves, their research, and their expression in ways that are meaningful to them. This makes space for multiple ways of knowing and representing knowledge, challenging rigid academic conventions and validating alternative epistemologies. In this way, metaphors themselves, and the exploration/application of metaphor as a learning process, can democratise the research process, making learning more accessible and meaningful for learners from diverse disciplinary backgrounds.

### Implications

This research offers significance across two central domains:

For RHD candidates or more novice researchers, it assists in articulating a structured, reflective, and creative approach to metaphor use. The paper provides a framework that can enhance the depth, clarity, and emotional resonance of scholarly work. The eight-step guide not only supports researchers in selecting and applying metaphors but also encourages critical engagement with the epistemological and ideological dimensions of their work.

For supervisors and academic teams, this paper has implications for both the practice and pedagogy of research. Supervisors can use the steps and reflective exercises to guide students in developing their scholarly voice, fostering creativity and navigating the emotional and intellectual challenges of the research journey. In coursework and workshops, the framework can be adapted to support learning in qualitative methods, critical theory, and academic writing.

## Conclusion

This paper has explored the multifaceted role of metaphor in RHDs, and indeed research more broadly, offering an eight-step framework to guide thoughtful and intentional application. Through theoretical grounding, practical examples, and reflective exercises, we have demonstrated how metaphor can illuminate complex ideas, evoke emotional resonance, and create coherence across a body of work. The use of metaphor requires careful consideration of audience, context, and purpose. Yet, when applied with intention, metaphor can serve as a bridge between abstract theory and lived experience, between researcher and reader and between academic discourse and public understanding. As research becomes increasingly interdisciplinary, creative, and socially engaged, the role of metaphor will only grow in significance. We encourage researchers, educators, and supervisors to embrace metaphor not only as a methodological tool but also as a pedagogical ally, one that fosters critical thinking, creativity, and connection.

**Key points for policy, practice and/or research**

- Metaphor is a powerful pedagogical strategy that enhances critical thinking, emotional engagement, and scholarly identity formation among nursing students and researchers. This paper has implications for nursing education, where metaphor can be used to scaffold complex concepts and support reflective learning.
- This paper has pragmatic and practical application for nurses (or indeed other candidates) engaging in higher research. An eight-step guide is introduced to help researchers apply metaphor intentionally and effectively. This framework supports the development of coherent narratives and deeper conceptual understanding, which can improve the quality and impact of nursing and health research.
- The paper highlights the role of metaphor in strengthening supervision relationships and guiding students through the emotional and intellectual challenges of research. Supervisors in nursing and health disciplines can use metaphor to foster creativity, epistemological awareness, and critical engagement.
- Metaphor is shown to be a valuable tool for translating complex health and social care issues into accessible narratives. This has policy implications, as metaphor can aid in advocacy, public engagement, and the dissemination of research findings to broader audiences, including policymakers and practitioners.

## References

[bibr1-17449871251403373] Abawi L (2013) Metaphors for, in and of education research ( Midgley W Trimmer K Davies A eds.). Cambridge: Cambridge Scholars Publishing.

[bibr2-17449871251403373] Alharbi AN (2023) Theoretical evolution of metaphor. Studies in Literature and Language 26: 1–8. DOI: 10.3968/13041.

[bibr3-17449871251403373] Aristotle (1902) The poetics ( Bywater I ,Trans.) Available at: http://www.authorama.com/book/the-poetics.html

[bibr4-17449871251403373] Barnard C (2025) The Attitudes Towards Non-suicidal Self-injury Held by Nurses Who Work in General Settings in Rural and Remote Australia. Australia: CQUniversity. DOI: 10.25946/28502597.v1.

[bibr5-17449871251403373] Byrne A-L Dhollande S Calleja P (2025) Articulating your research focus via a capability statement: Professional development for early career researchers. Journal of Research in Nursing. DOI: 10.1177/17449871241307139 PMC11907647 40093816

[bibr6-17449871251403373] Byrne A-L (2022) Person-centred Care as a technology of compliance: A critical investigation of how nurse navigators care for people with complex conditions. Doctoral Thesis [CQUniversity]. Available at: https://acquire.cqu.edu.au/articles/thesis/Person-centred_care_as_a_technology_of_compliance_A_critical_investigation_of_how_nurse_navigators_care_for_people_with_complex_conditions/23632461?file=41468349

[bibr7-17449871251403373] Cardona M (2020) Defending Qualitative Research : Design, Analysis and Textualization, 1st edn. London: Routledge.

[bibr8-17449871251403373] Carpenter J (2008) Metaphor in Qualitative Research: Shedding light or casting shadows. Research in Nursing and Health 31: 274–282. DOI: 1002/nur.20253. 18196583 10.1002/nur.20253

[bibr9-17449871251403373] Carrol L (ed.1018) (1865) Alice in Wonderland. Wordsworth Editions. (Original work published 1865).

[bibr10-17449871251403373] Carston R (2018) Figurative language, mental imagery, and pragmatics. Metaphor and Symbol 33: 198–217. DOI: 1080/10926488.2018.1481257.

[bibr11-17449871251403373] Declercq J van Poppel L (2024). Metaphors in interaction: Reusing, developing and resisting metaphors of illness, the body and medical treatment in chronic pain consultations. Journal of Pragmatics 221: 168–182. DOI: 10.1016/j.pragma.2024.01.001.

[bibr12-17449871251403373] Douglas JK (2018) Shakespeare’s World: The Tragedies: A Historical Exploration of Literature. Greenwood, p. xvii https://search.ebscohost.com/login.aspx?direct=true&AuthType=sso&db=e000xww&AN=1823363&scope=site (accessed 29 April 2025).

[bibr13-17449871251403373] Durkee DA (2011) Teaching with metaphor: The case of Alice in Gaap land. Academy of Educational Leadership Journal 15: 1–19.

[bibr14-17449871251403373] Edwards-Groves CJ Ronnerman K (2021) Generative Leadership. Singapore: Springer.

[bibr15-17449871251403373] Expósito Ropero N Soares da Silva A (2024) Phenomenology and cognitive linguistics in dialogue: A review of Ortega y Gasset’s theory of emotive gesture as metaphor. The Southern Journal of Philosophy 62: 374–390. DOI: 10.1111/sjp.12555

[bibr16-17449871251403373] Fetterman AK Evans ND Covarrubias JJ (2020) On the Interpersonal Function of Metaphor Use Daily Metaphor Use Fluctuates With Empathy and Perspective Taking. Social Sciences, 52(1). 10.1027/1864-9335/a000431

[bibr17-17449871251403373] Filipczuk-Rosinska S (2006) Analysing metaphorical political discourse in the L2 academic classroom. Social and Behaviousal Sciences 228: 329–334. DOI: 10.1016/j.sbspro.2016.07.049

[bibr18-17449871251403373] Foucault M (2007) Discipline and punish: The birth of the prison. In Lawrence B Karim A (eds) On Violence: A Reader. New York, NY: Duke University Press, pp. 445–471. DOI: 10.1515/9780822390169-058.

[bibr19-17449871251403373] Foucault M (2008) “The Birth of Bio-Politics”–Michel Foucault’s Lecture at the Collège de France on Neo-Liberal Governmentality. Economy and Society 30: 190–207.

[bibr20-17449871251403373] Frick BL Brodin EM (2020) A return to Wonderland: Exploring the links between academic identity development and creativity during doctoral education. Innovations in Education and Teaching International 57: 209–219.

[bibr21-17449871251403373] Garello S (2024) The enigma of metaphor. In Garello S (eds.) The Enigma of Metaphor: Philosophy, Pragmatics, Cognitive Science. Cham: Springer Nature Switzerland, pp. 1–17.

[bibr22-17449871251403373] Garlitz D Zompetti J (2021) Critical theory as post-Marxism: The Frankfurt School and beyond. Educational Philosophy and Theory 55: 141–148. DOI: 10.1080/00131857.2021.1876669.

[bibr23-17449871251403373] Goatly A (2006) Ideology and metaphor. English Today 87: 25–39. DOI: 10.1017/S0266078406003051

[bibr24-17449871251403373] Government releases final report of the Royal Commission into Aged Care: 7.30. (2021, March 2). ABC News. [video file] Available at: https://www.youtube.com/watch?v=kuo0QuuMG5k

[bibr25-17449871251403373] Guilherme A Souza de Freitas AL (2018) Discussing education by means of metaphors. Educational Philosophy and Theory 50: 947–956. DOI: 10.1080/00131857.2016.1198250

[bibr26-17449871251403373] Hanić J Pavlović T Jahić Jašić A (2018) Journey through the writing process: Metaphors of thesis writing experience. Explorations in English Language and Linguistics 6: 163–179.

[bibr27-17449871251403373] Harvey C Hegney D Sobolewska A , et al. (2019) Developing a community-based nursing and midwifery career pathway – A narrative systematic review. PLos One 14:1–16. DOI: 10.1371/journal.pone.0211160 PMC6438448 30921338

[bibr28-17449871251403373] Ivie SD (2022) Metaphor: Key to critical and creative thinking. Journal of Thought 56: 55–77.

[bibr29-17449871251403373] Jamrozik A McQuire M Cardillo ER , et al. (2016) Metaphor: bridging embodiment to abstraction. Psychonomic Bulletin & Review 23: 1080–1089. DOI: 10.3758/s13423-015-0861-0 27294425 PMC5033247

[bibr30-17449871251403373] Jensen T (2019) What is a Metaphor? Oregon State University. https://liberalarts.oregonstate.edu/wlf/what-metaphor

[bibr31-17449871251403373] Johnson MI Hudson M Ryan CG (2023) Perspectives on the insidious nature of pain metaphor: we literally need to change our metaphors. Frontline Pain Research 15: 1–13. DOI: 10.3389/fpain.2023.1224139 PMC10540619 37781218

[bibr32-17449871251403373] Kelly F (2011) “Cooking together disparate things”: The role of metaphor in thesis writing. Innovations in Education and Teaching International 48: 429–438. DOI: 1080/14703297.2011.617088.

[bibr33-17449871251403373] Kövecses Z (2020) Extended Conceptual Metaphor Theory. Cambridge: Cambridge University Press. DOI: 10.1017/9781108859127.

[bibr34-17449871251403373] Lakoff G (1992) The contemporary theory of metaphor. Available at: https://terpconnect.umd.edu/~israel/lakoff-ConTheorMetaphor.pdf

[bibr35-17449871251403373] Lakoff G Johnson M (1980) Metaphors We Live By. Chicago: The University of Chicago Press.

[bibr36-17449871251403373] Lemke T (2001) “The birth of bio-politics”: Michel Foucault’s lecture at the Collège de France on neo-liberal governmentality. Economy and Society 30: 190–207. DOI: 10.1080/03085140120042271.

[bibr37-17449871251403373] Lit Charts (n.d.) Ozymandias Summary & Analysis by Percy Bysshe Shelley. Lit Charts. Available at: https://www.litcharts.com/poetry/percy-bysshe-shelley/ozymandias

[bibr38-17449871251403373] Lubart TI Getz I (1997) Emotion, metaphor, and the creative Process. Creativity Research Journal 10(4): 285–301. DOI: 10.1207/s15326934crj1004_1.

[bibr39-17449871251403373] Lynch HL Fisher-Ari TR (2017) Metaphor as pedagogy in teacher education. Teaching and Teacher Education 66: 195–203.

[bibr40-17449871251403373] Mainey L Essex R Gurnett P , et al. (2025) Stirring the Sleeping Giant? An evaluation of a planetary health political action sequential simulation for nursing students. Nursing Inquiry 32: e70017. 10.1111/nin.70017 PMC11973622 40190272

[bibr41-17449871251403373] Malone M (2023) Re-imagining the research article: Social-semiotic signposts and the potential for radical co-presence in the scholarly literature. International Journal of Community Research and Engagement 16. Available at: https://epress.lib.uts.edu.au/journals/index.php/ijcre/article/view/8606/8206

[bibr42-17449871251403373] Manhas KP Oberle K (2015) The ethics of metaphor as a research tool. Research Ethics 11: 42–51.

[bibr43-17449871251403373] McIntosh N (2015) A passion for research: Senior nurse Nichole McIntosh describes how becoming a PhD research student has not only enhanced her career but has proven a life-changing experience. Nursing Standard 29: 63–63. DOI: 10.7748/ns.29.32.63.s45 25850511

[bibr44-17449871251403373] McLeod S (2024) Freud’s theory of the unconscious mind. Psychology, Freudian Psychology. Available at: https://www.simpypsychology.org/unconscious-mind.html

[bibr45-17449871251403373] McNaughton SL Harvey C Baldwin A (2022) The map to women centred care for a woman experiencing a complex pregnancy. Australian Midwifery News. Available at: https://search.informit.org/doi/pdf/10.3316/informit.819640104647616

[bibr46-17449871251403373] Mouraz A Pereira AV Monteiro R (2013) The use of metaphors in the processes of teaching and learning in higher education. International Online Journal of Educational Sciences 5: 99–110.

[bibr47-17449871251403373] Mulvogue J (2024) Machinations of restraint in residential aged care facilities. A critical discourse analysis. Doctoral Thesis, CQUniversity. Available at: https://acquire.cqu.edu.u/articles/thesis/The_machinatioresearchfindingsmeaningful.Canns_of_restraint_use_in_residntial_aged_care_A_critical_discourse_analysis/25483873?file=45289756

[bibr48-17449871251403373] Povinelli A (2011) Economies of Abandonment. Social Belonging and Endurance in Late Liberalism. Duke University Press.

[bibr49-17449871251403373] Redden SM (2017) Metaphor analysis. In Matthes J Davis CS Potter RF (eds) The International Encyclopedia of Communication Research Methods. DOI: 10.1002/9781118901731.iecrm0154

[bibr50-17449871251403373] Reddy MJ (1979) The conduit metaphor: A case of frame conflict in our language about language. In Ortony A (Ed.), Metaphor and thought. Cambridge: Cambridge University Press, pp. 284–310.

[bibr51-17449871251403373] Scobie M Lee B Smyth S (2021) Braiding together student and supervisor aspirations in a struggle to decolonize. Organization 28: 857–875.

[bibr52-17449871251403373] Semino DZ Demmen J (2018) An integrated approach to metaphor and framing in cognition, discourse, and practice, with an application to metaphors for cancer. Applied Linguistics 39: 625–645. DOI: 10.1093/applin/amw028.

[bibr53-17449871251403373] Shakespear W (1623) As you like it. The Folio (first printed version). Act 2, p. 7. Available at: https://internetshakespeare.uvic.ca/doc/AYL_F1/scene/2.7/index.html

[bibr54-17449871251403373] Shelley P.B . (1817) ‘Ozymandias’. In Reiman D.H. Fraistat N . (eds.) The Norton Anthology of English Literature: The Romantic Period. New York: W.W. Norton, pp. 742–743.

[bibr55-17449871251403373] Stanley BL Zanin AC Avalos BL , et al. (2021) Collective emotion during collective trauma: A metaphor analysis of the COVID-19 pandemic. Qualitative Health Research 31: 1890–1903. DOI: 10.1177/10497323211011589. 33980096

[bibr56-17449871251403373] Steele R Baird J Davies B (2022) Using metaphors to make research findings meaningful. Canadian Journal of Nursing Research 54: 99–100. DOI: 10.1177/08445621221085555. PMC9109576 35238223

[bibr57-17449871251403373] Steen GJ (2007) Foundations. In Steen G. J . (eds.) Finding Metaphor in Grammar and Usage. 26–64. John Benjamins Publishing Company.

[bibr58-17449871251403373] Steen G (2011) The contemporary theory of metaphor – now new and improved! Review of Cognitive Linguistics 9: 26–64. DOI: 10.1075/rcl.9.1.03ste.

[bibr59-17449871251403373] Taylor C Dewsbury BM (2018) On the problem and promise of metaphor use in science and science communication. Science Communication 19. DOI: 1128/jmbe.v19i1.1538. 10.1128/jmbe.v19i1.1538 PMC5969428 29904542

[bibr60-17449871251403373] Tenniel J (1967) Alice’s Adventures in Wonderland. London: McMilliam and Co.

[bibr61-17449871251403373] Tourangeau R Sternberg RJ (1982) Understanding and appreciating metaphors. Cognition 11: 203–244. DOI: 10.1016/0010-0277(82)90016-6. 7199412

[bibr62-17449871251403373] Ventalon G Erjavec G Tijus C (2023) A review of processing and analysing visual metaphors in psychology. European Review of Applied Psychology 74. 10.1016/j.erap.2022.100836

[bibr63-17449871251403373] Yildiz M Dede D (2025) The use and misuse of metaphors in e-government studies. Government Information Quarterly, 42: 102067.

[bibr64-17449871251403373] Waring M Evans C (2015) Making sense of pedagogy. In Waring M Evans C . (eds.) Understanding Pedagogy, 1st edn. Abingdon, Oxford: Routledge, pp. 26–30. DOI: 10.4324/9781315746159-2

[bibr65-17449871251403373] Wodak R (2014) Critical discourse analysis. In Flowerdew J Richardson J.E . (Eds.). The Routledge companion to English Studies. London: Routledge, pp. 302–316.

